# Hemisection: A Different Approach From Extraction

**DOI:** 10.7759/cureus.29410

**Published:** 2022-09-21

**Authors:** Prachi Taori, Pradnya P Nikhade, Joyeeta Mahapatra

**Affiliations:** 1 Department of Conservative Dentistry and Endodontics, Sharad Pawar Dental College, Datta Meghe Institute of Medical Science, Wardha, IND; 2 Department of Conservative Dentistry and Endodontics, Sharad pawar Dental College, Datta Meghe Institute of Medical Science, Wardha, IND

**Keywords:** endodontic surgery, appropriate case selection, mandibular molar, root caries, hemisection

## Abstract

Hemisection is the sectioning of teeth with multiple roots, the removal of the damaged root and its associated crown piece, and the preservation of the healthy root (with crown). When periodontal, resorption, perforation, or caries damage is limited to one root and the other root is still largely healthy, this treatment option may be taken into account. The right case selection is the most important component in determining the long-term success in such cases. This case report details the hemisection of a mandibular molar with root caries, followed by appropriate restoration.

## Introduction

Advanced dental therapeutic techniques have made it possible to keep teeth that were previously thought to be irreparable. These treatment methods use a multidisciplinary approach. One such therapy is hemisection, which integrates principles from prosthodontics, oral surgery, endodontics, periodontics, and restorative dentistry [1]. A multi-rooted tooth undergoes surgery in which the healthy section of the crown and its associated root are left in place at the level of the furcation, retaining the tooth's integrity within the socket. One-half of the crown and the associated unrestorable root are also removed. The most important consideration in such instances that needs a comprehensive pre-operative assessment in prosthodontics, endodontics, and periodontics is case selection [2].

The indications for tooth hemisection, according to Weine [3] are Periodontal Indications: a) Only one root of a tooth with several roots suffers from severe vertical bone loss. b) Complete destruction of the furcation. c) The roots of adjacent teeth are too close to one another, making it difficult to maintain proper cleanliness in close quarters. d) Serious root exposure as a result of dehiscence.

Endodontic and Restorative Indications: a) Prosthetic failure of abutments within a splint: If a single or multiple-rooted tooth is periodontally affected within a fixed bridge, the root of the concerned tooth is excised in place of removing the complete bridge if the remaining abutment support is adequate. b) Endodontic failure: Hemisection is helpful when a pulp chamber's floor or a root's pulp canal of a tooth with endodontic involvement has been perforated and cannot be instrumented. c) Vertical fracture of a single root: Vertical fracture has no chance of recovery. Hemisection can be performed if a vertical fracture crosses one root but leaves the other root undamaged. d) Extremely destructive process: This can happen as a result of endodontic therapy, trauma, significant root perforation, furcation, or subgingival caries.

Contraindications: a) As alternatives to hemisection, there are strong neighboring teeth that can serve as bridge abutments. b) Inoperable root canals should be kept. c)Fusion of the roots makes separation impossible. d) Non-strategic teeth.

## Case presentation

A 56-year-old male patient reported to the Department of Conservative Dentistry and Endodontics, Datta Meghe Institute of Medical Science, Wardha, India, with the chief complaint of pain in the lower left back region of the jaw for one month. On clinical examination, disto-proximal caries was seen was 36, which was tender on percussion and mesio-proximal caries with 37. The patient's medical history was non-contributory. Oral hygiene maintenance of the patient was poor for which he was advised oral prophylaxis. Lymph nodes in the head and neck region were not palpable. The chair-side examination was conducted. Electric pulp testing gave a delayed response for both 36 and 37.

**Figure 1:**
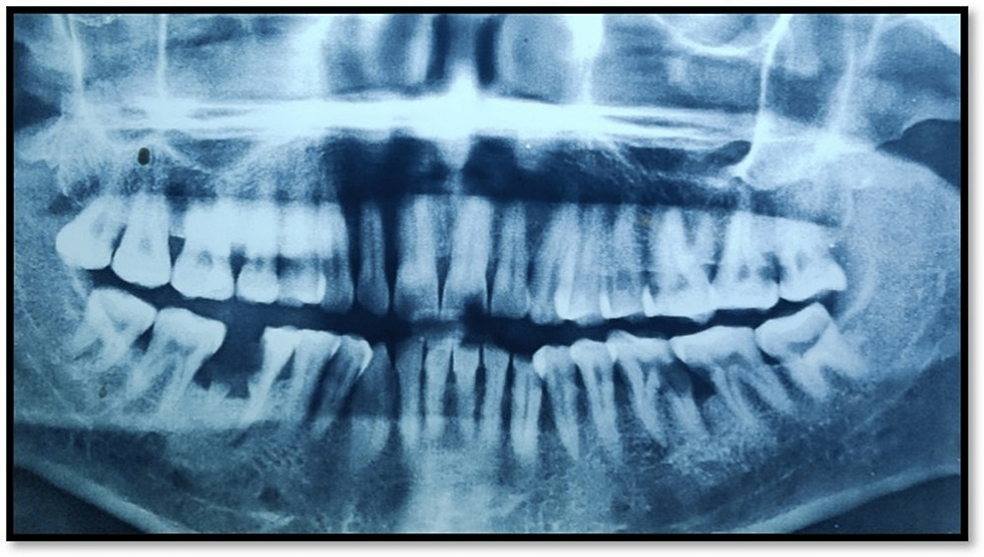
OPG showing disto-proximal caries with 36 OPG - Orthopantomogram

Orthopantomogram (Figure 1) showed disto-proximal caries with 36 which involved enamel, dentin and pulp, which also extended to the radicular portion of 36. In the apical portion, PDL widening was seen associated with 36. Mesio-proximal caries with 37, which involved enamel and dentin. Teeth were diagnosed as irreversible pulpitis with chronic apical periodontitis with 36 and irreversible pulpitis with 37.

**Figure 2:**
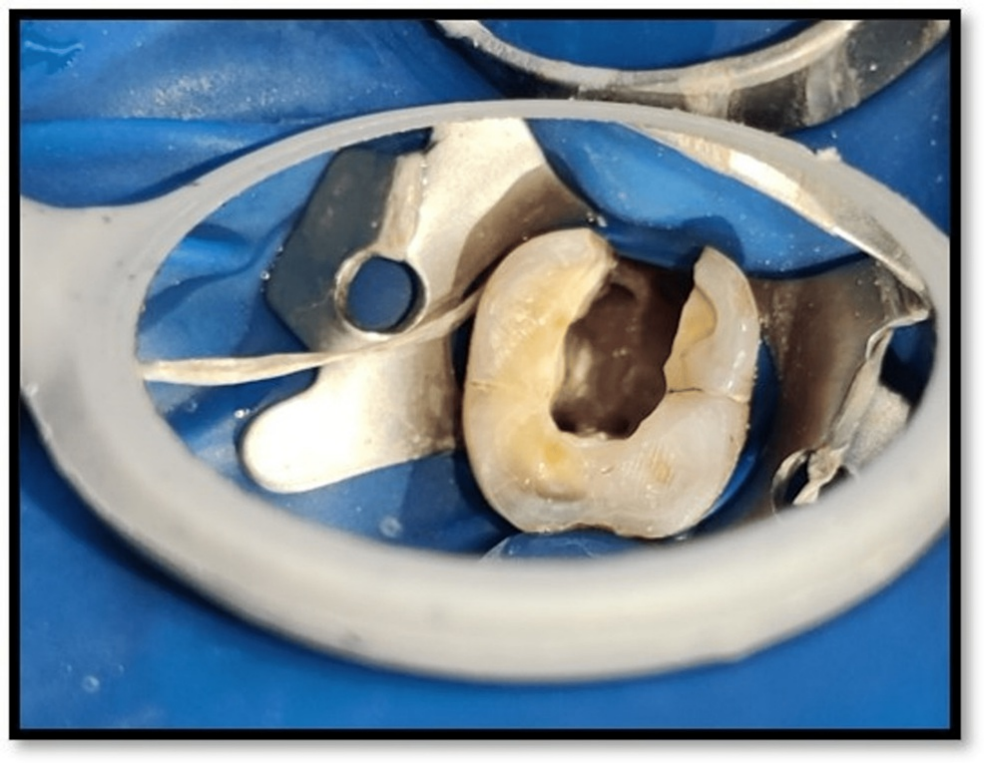
Access opening with 36

**Figure 3:**
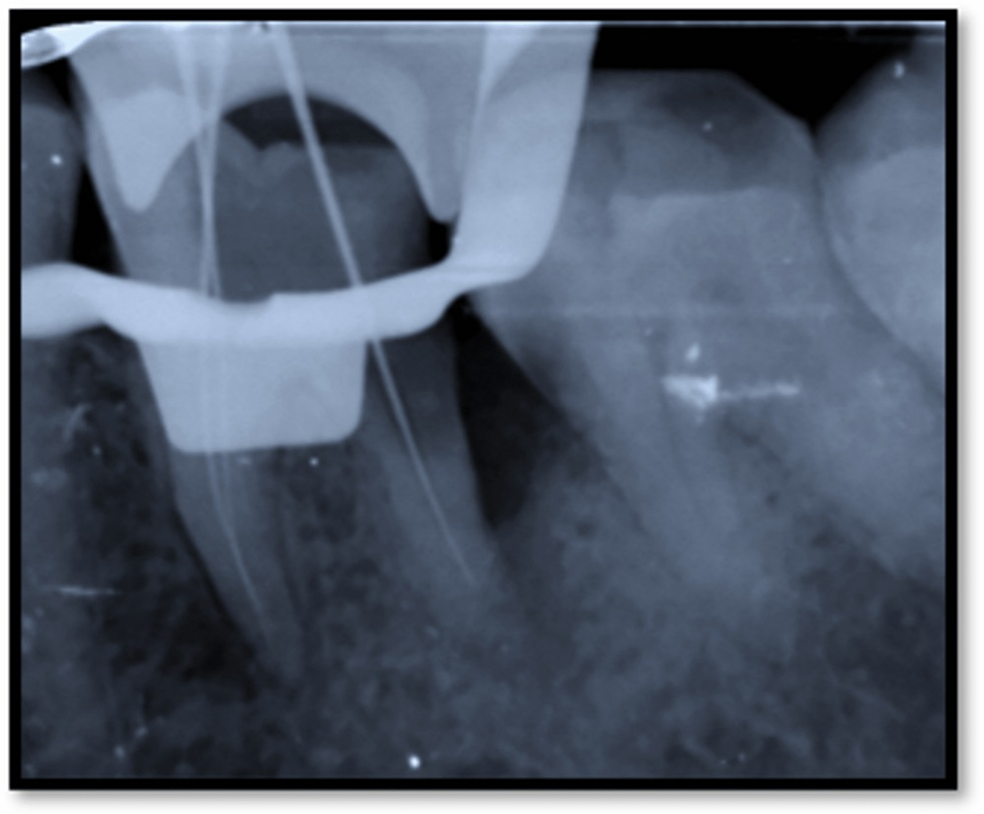
Working length with 36

The treatment plan was explained to the patient and his inform consent was obtained. Oral prophylaxis was done out before commencement of the root canal procedure. Rubber dam isolation was done. Access opening was carried out using round bur (BR- 45, Mani, Japan) and safe end bur (EX 24, Mani, Japan) followed by pulp extirpation with 36 (Figure 2). Three canals were located in 36, i.e., Mesiobuccal (MB), Mesiolingual (ML), and Disto (D). Working length was determined using radiograph and electronic apex locator (J Morita Root Zx Mini, Tokyo, Japan). Working length with MB=18mm, ML=19mm, and D=17mm (Figure 3). Canals were irrigated with 3% sodium hypochlorite (Parcan, Septodont, India) and 0.9% normal saline for remove of remaining pulp tissue and debris. Biomechanical preparation was carried out up to F2 protaper in all the t canals. Then calcium hydroxide (Prime RC Cal, India) dressing was placed within canal and a temporary pack (T-fill, Belgium) was given. The patient was recalled for the second visit.

**Figure 4:**
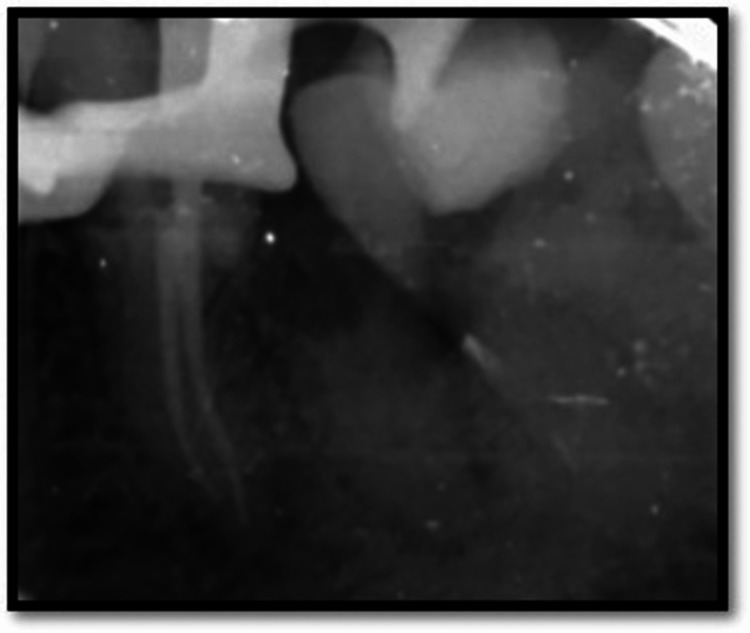
Obturation of mesial root of 36

**Figure 5:**
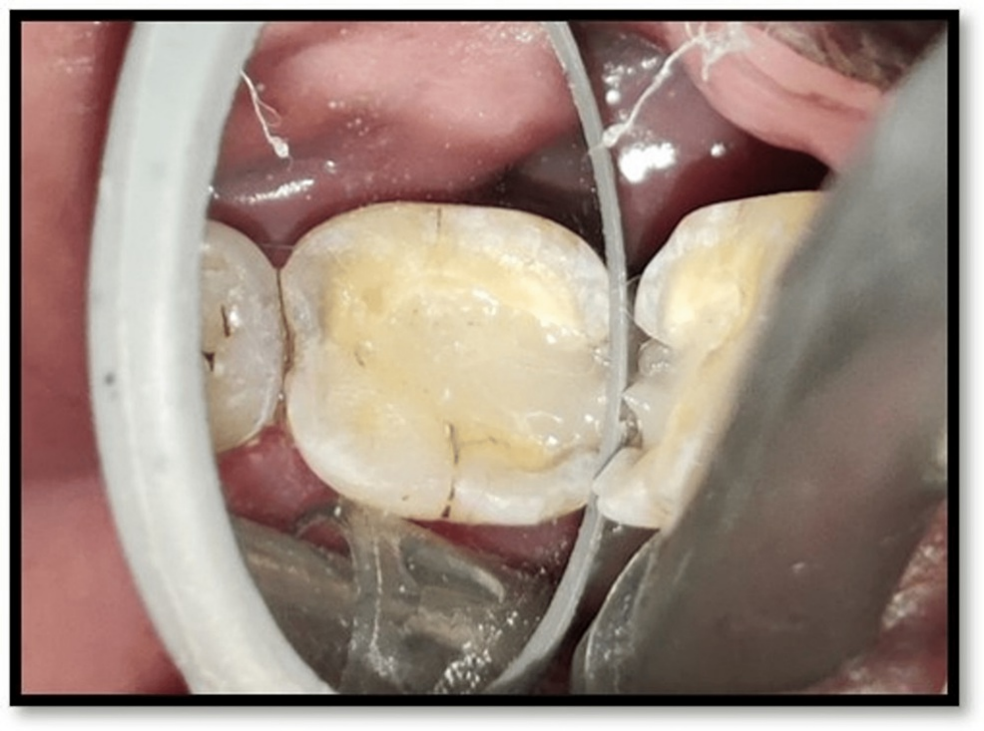
Post-endodontic composite restoration with 36

On the second visit, a temporary dressing was removed, and canal was irrigated using saline and an endoactivator to remove calcium hydroxide from the canal. Gutta-percha master cones were selected and obturation was done in the mesial the root of 36 (Figure 4). Post-endodontia full thickness was done using composite with 36 (Figure 5).

**Figure 6:**
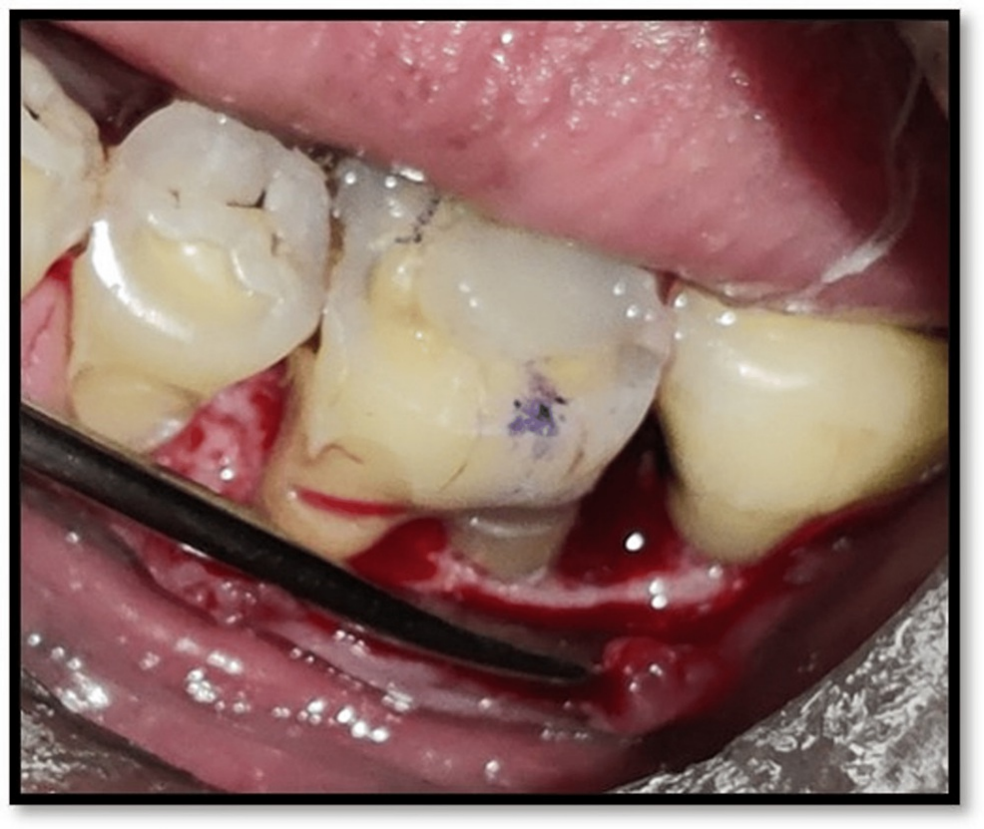
Flap reflection from second premolar to second molar

**Figure 7:**
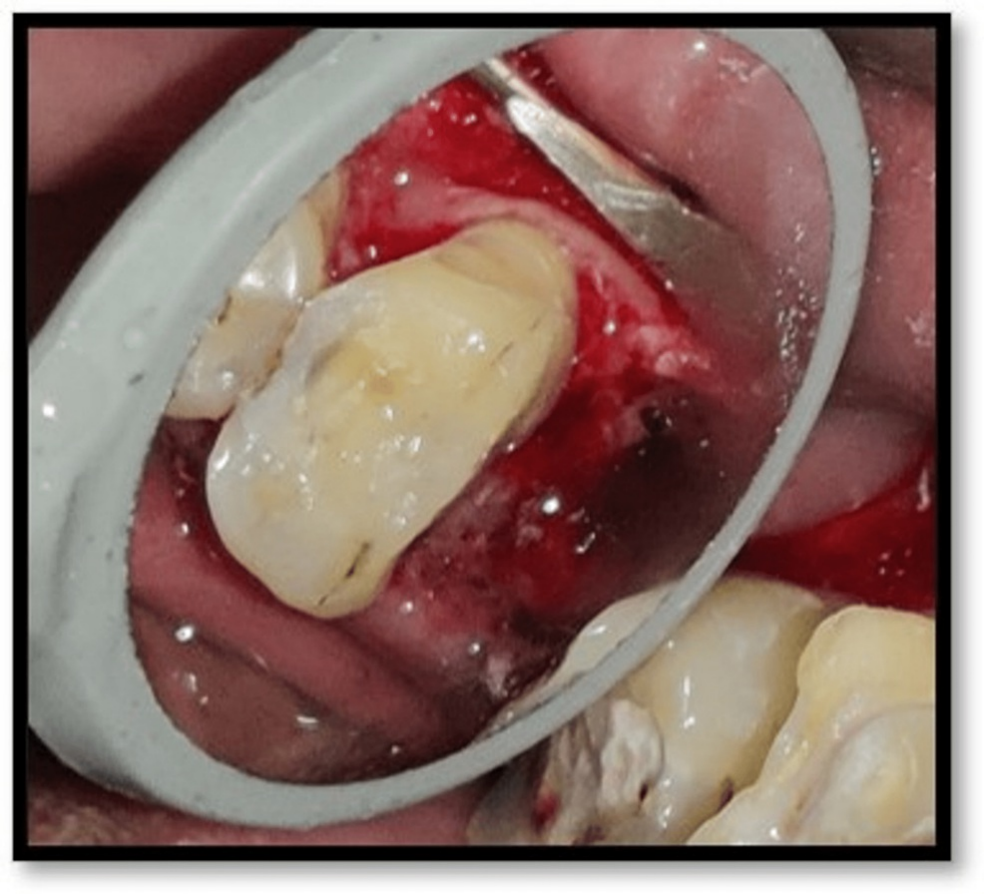
Placement of PRF in the extracted root socket

**Figure 8:**
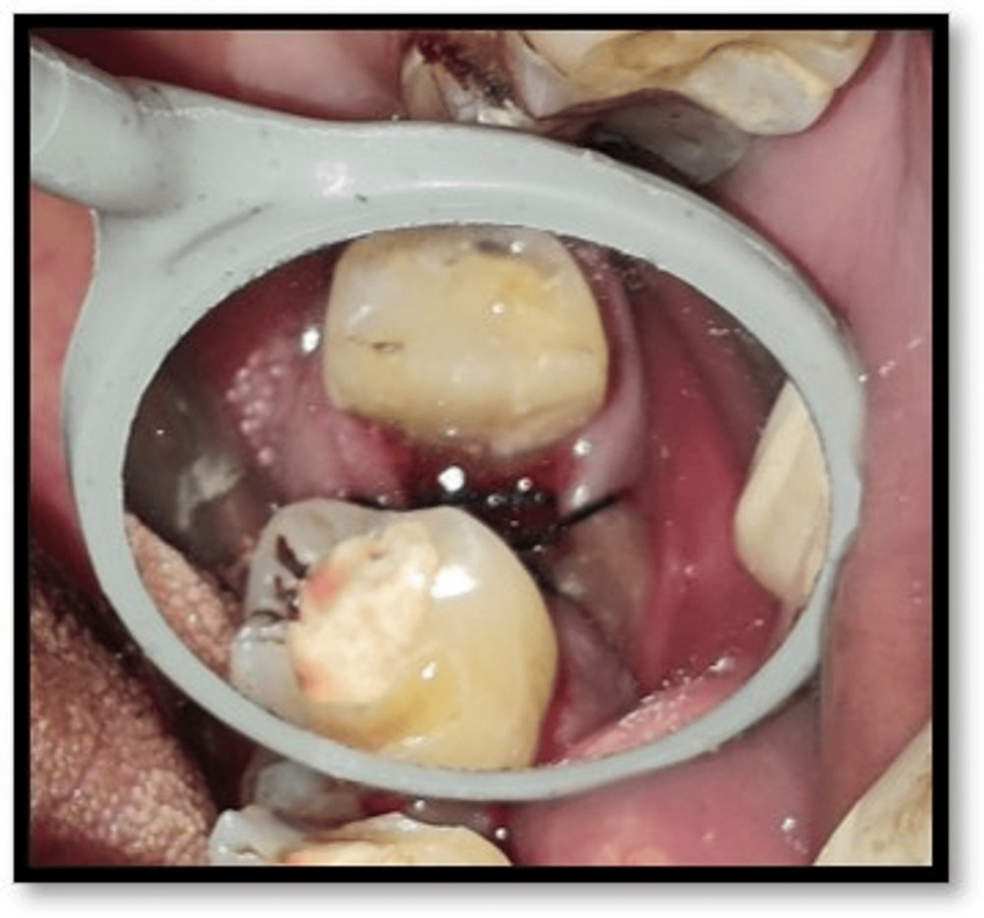
Suturing with figure of 8 technique

**Figure 9:**
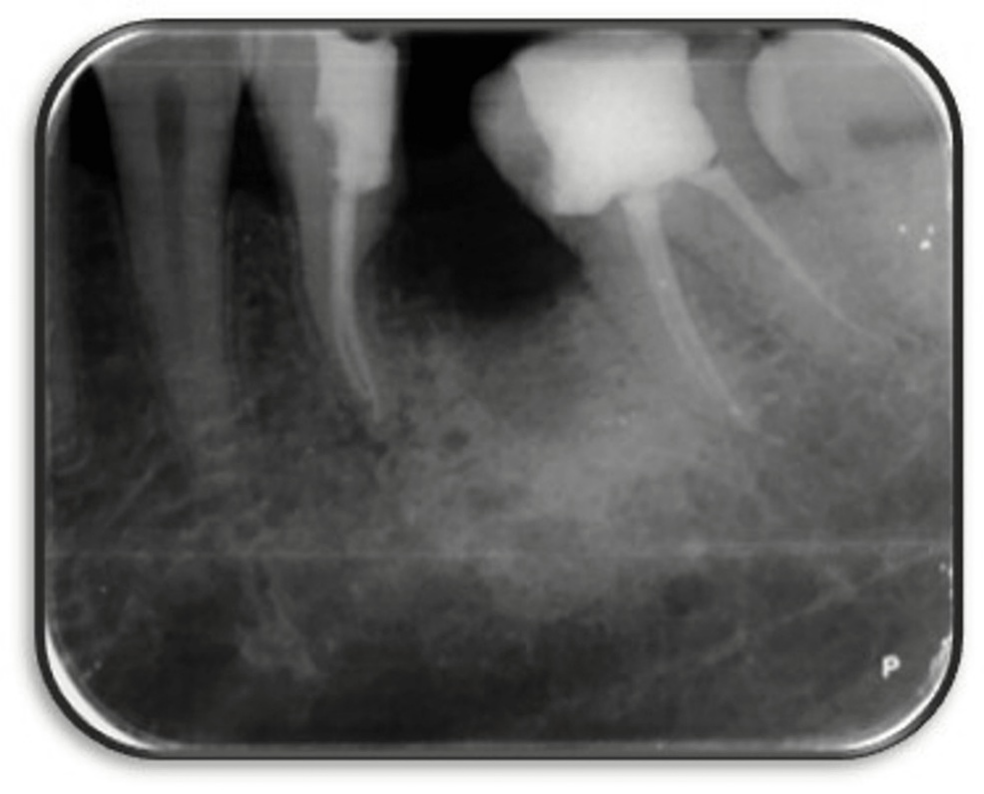
Post-operative radiograph

**Figure 10:**
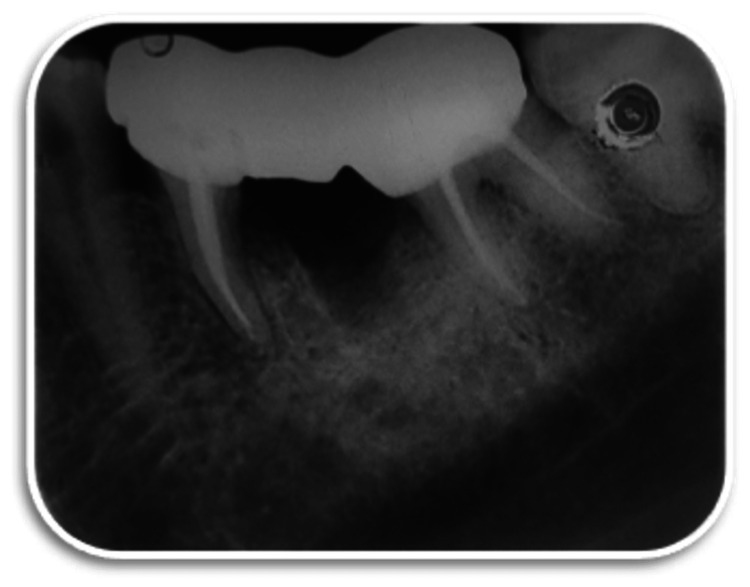
Three-month follow-up radiograph

On third visit, under a local anesthesia, full thickness flap was reflected after giving a crevicular incision from second premolar to second molar (figure 6). The vertical cut method was used to resect the crown. A long shank tapered fissure carbide bur was used to make vertical cut toward the bifurcation area. A fine probe was passed through the cut to ensure separation. The distal root of 36 was extracted. Socket was irrigated adequately with saline to remove bony chips. PRF was prepared using patient’s blood. Prepared PRF was placed in the extracted root socket (Figure 7). Following this suturing was done using 3-0 Vicryl suture using figure of 8 technique (Figure 8). Post-operative radiograph is shown in Figure 9. Figure 10 depicts radiographic image after three months follow-up.

## Discussion

A useful alternative method to save multi-rooted teeth that have been recommended for extraction is hemisection, often known as root amputation. If the right cases are chosen, it is a reasonably straightforward, conservative, affordable treatment with good prospects of success [4]. The clinician's decision to select a case for hemisection is influenced by a variety of circumstances. These can be categorized into three categories.

**Table 1:** Categories of case selection

Categories of case selection
Local considerations, such as tooth anatomy, mobility, crown-to-root ratio, degree of attachment loss, interaction between the inter- and intra-arch teeth, and strategic dental value for retention or removal.
Patient factors like health of a patient, importance of the tooth to the patient, costs, and time factor.
Clinical aspects include a careful case selection process, diagnostic and treatment planning abilities, knowledge of available therapy alternatives, and clinical insight or service delivery expertise.

According to Park et al. [5], hemisection is a dependable treatment choice for molars with a dubious prognosis since it will keep the teeth without a noticeable bone loss for a protracted length of time if the patient maintains excellent dental hygiene. Additionally, it is important to evaluate the root's accessibility for simple separation and the root's remaining root's bone support. With a prognosis comparable to any tooth undergoing endodontic therapy, this procedure provides a reliable therapeutic choice.

The overall survival rate of a large number of root-resected molars by Yuh et al. in a retrospective study was found to be 91.1%. [6]. A survival rate of nearly 93% over a 10-year follow-up was reported in another study by Carnevale et al. in cases where hemisection was performed for the management of furcated molars [7].

For the quality of the resections, Newell [8] assessed 62 patients' 70 root-resected molars. When subgingival caries, residual roots, or ledges were present in 21 (30%) of the resections, those procedures were deemed flawed. Maxillary molar failure rates were higher (33.3%) than mandibular molar failure rates (22.7%). A positive outcome is frequently eliminated by deterioration following therapy. Failure of endodontic therapy can result from a variety of factors. Additionally, if the tooth's root support has lost some of it, a restoration will be necessary to allow it to operate independently or act as an abutment for a bridge or splint.

On 34 resected molars, Buhler [9] found a 32% failure rate at 10 years. Again, endodontic pathology and root fracture were the primary causes of failure, whereas only one tooth was taken because of periodontal disease. In a follow-up of three to 10 years, Blomlof et al. discovered the same failure rate [10].

Shafiq hemisected the tooth and mesial root was removed along with the dislodged part [11], and a three-unit bridge combining the hemisected root and adjacent second premolar was inserted which is successfully in service for more than a year. He concluded that the retention of a part of a tooth seems to extend the life of a prosthesis; the patient certainly deserves the option of hemisection or root amputation rather than extraction.

Kumar performed a hemisection procedure on a 45-year-old male patient [12]. It had a persistent chronic sinus tract associated with a periodontal pocket in relation to an endodontically treated and crowned lower right first molar. Following a provisional diagnosis of vertical root fracture in relation to mesial root of 46, exploratory surgery was done. The mesial root was extracted. Socket preservation was done by grafting the extraction site with a mixture of the demineralized freeze-dried bone allograft.

But if there are poor margins or poor physiologic shape on non-occlusal surfaces, a restoration can worsen periodontal disease. Additionally, a tooth may be more vulnerable to occlusion-related damage and eventually, hemisection fails if the occlusal contact area is incorrectly formed.

## Conclusions

Following a firm set of criteria for case selection and specific endodontic, surgical, and restorative guidelines hemisection can prove to be a reliable treatment option in saving molar teeth once considered non-restorable. It has also been suggested that hemisection should be considered before every molar extraction, as it offers successful long-term results.
